# Decursinol Angelate Mitigates Sepsis Induced by Methicillin-Resistant *Staphylococcus aureus* Infection by Modulating the Inflammatory Responses of Macrophages

**DOI:** 10.3390/ijms222010950

**Published:** 2021-10-11

**Authors:** Seongwon Pak, Bikash Thapa, Keunwook Lee

**Affiliations:** 1 Department of Biomedical Science, Hallym University, Chuncheon 24252, Korea; seongwon@hallym.ac.kr; 2 Institute of Bioscience and Biotechnology, Hallym University, Chuncheon 24252, Korea; bikash@hallym.ac.kr

**Keywords:** decursinol angelate, sepsis, methicillin-resistant *Staphylococcus aureus*, macrophage, NF-κB, Akt

## Abstract

The herbal plant *Angelica gigas* (*A. gigas*) has been used in traditional medicine in East Asian countries, and its chemical components are reported to have many pharmacological effects. In this study, we showed that a bioactive ingredient of *A. gigas* modulates the functional activity of macrophages and investigated its effect on inflammation using a sepsis model. Among 12 different compounds derived from *A. gigas*, decursinol angelate (DA) was identified as the most effective in suppressing the induction of TNF-α and IL-6 in murine macrophages. When mice were infected with a lethal dose of methicillin-resistant *Staphylococcus aureus* (MRSA), DA treatment improved the mortality and bacteremia, and attenuated the cytokine storm, which was associated with decreased CD38^+^ macrophage populations in the blood and liver. In vitro studies revealed that DA inhibited the functional activation of macrophages in the expression of pro-inflammatory mediators in response to microbial infection, while promoting the bacterial killing ability with an increased production of reactive oxygen species. Mechanistically, DA treatment attenuated the NF-κB and Akt signaling pathways. Intriguingly, ectopic expression of an active mutant of IKK2 released the inhibition of TNF-α production by the DA treatment, whereas the inhibition of Akt resulted in enhanced ROS production. Taken together, our experimental evidence demonstrated that DA modulates the functional activities of pro-inflammatory macrophages and that DA could be a potential therapeutic agent in the management of sepsis.

## 1. Introduction

Upon pathogen infection, the innate immune system orchestrates an immediate early response to the invading microbe. Macrophages are a central player in innate immunity and trigger the inflammatory cascade by phagocytosing the microbes and secreting pro-inflammatory factors, such as IL-1β, IL-6, and TNF-α [1]. The local milieu of cytokines and chemokines, and the surface molecules on macrophages, such as class II MHC, and costimulatory ligands instruct adaptive immunity to shape an appropriate response against the pathogens, which includes type 1, type 2, and type 17 T-cell responses [2]. While evoking inflammation, macrophages are also involved in curtailing inflammation and repairing damaged tissues, especially in the resolution phase [3]. These macrophages express anti-inflammatory cytokines (IL-10), immunomodulatory enzymes (arginase-1), growth factors (VEGF-A), and matrix metalloproteinases [4]. In this context, the spatiotemporal balance among the functional states of macrophages should be tightly regulated to maintain tissue homeostasis; otherwise, it leads to inflammatory diseases, including arthritis, colitis, and sepsis.

Upon infection with a pathogen such as *Staphylococcus aureus*, macrophages are responsible for the elimination of the invading microbe and the inflammatory responses against the infection. However, deregulated activation of macrophages and the resulting upregulation of pro-inflammatory mediators, such as TNF-α, contribute to the pathogenesis of sepsis [5]. Antibiotics have been used to manage sepsis induced by bacterial infection, but the emergence of antibiotic-resistant strains has become a major global healthcare problem. Indeed, methicillin-resistant *S. aureus* (MRSA) is one of the major pathogens found in hospitals, and causes bacteremia with a poor outcome, leading to fatal sepsis [6]. Thus, approaches to control excessive inflammation are urgently needed for a therapeutic option to treat sepsis in combination with antibiotics.

*Angelica gigas,* also called Korean angelica, has been used as a traditional East Asian medicine in China and Korea. The roots of the plant are regarded as the so-called ‘female ginseng’ due to a beneficial effect on gynecologic health. Bioactive ingredients of the roots have been studied, in order to evaluate their potential to improve many disease conditions, including inflammatory diseases and cancer [7,8]. Pyranocoumarins, decursin, and its structural isomer decursinol angelate (DA) are the most abundant compounds of the ethanol extracts of *A. gigas* and are reported to have anti-inflammatory activities in several cancer cell lines [9,10]. In vivo studies using arthritis and asthma animal models have implied the immunomodulatory potential of decursin or DA [11,12], but it is unclear which part(s) of the immune system is affected at the cellular level. Although other studies have reported the anti-inflammatory activity of decursin or DA, using monocyte/macrophage cell lines (e.g., RAW264.7 and THP-1 cells) [13,14], how the functional activity of macrophages is regulated in a setting of inflammatory conditions remains to be determined.

In this study, we screened the immunomodulatory components of *A. gigas* and identified DA as the most prominent in suppressing the inflammatory responses of mouse primary macrophages. In a sepsis model using MRSA, we observed the beneficial effect of DA on septic symptoms. Intriguingly, DA attenuated the induction of pro-inflammatory cytokines upon bacterial infection, while promoting the bacterial killing ability of the macrophages. Furthermore, biochemical and rescue experiments revealed that DA modulates the NF-κB and Akt signaling pathways in relation to the functional activities of macrophages. 

## 2. Results

### 2.1. MRSA Infection Activates Macrophages Associated with the Pro-Inflammation Phenotype

**Figure 1:**
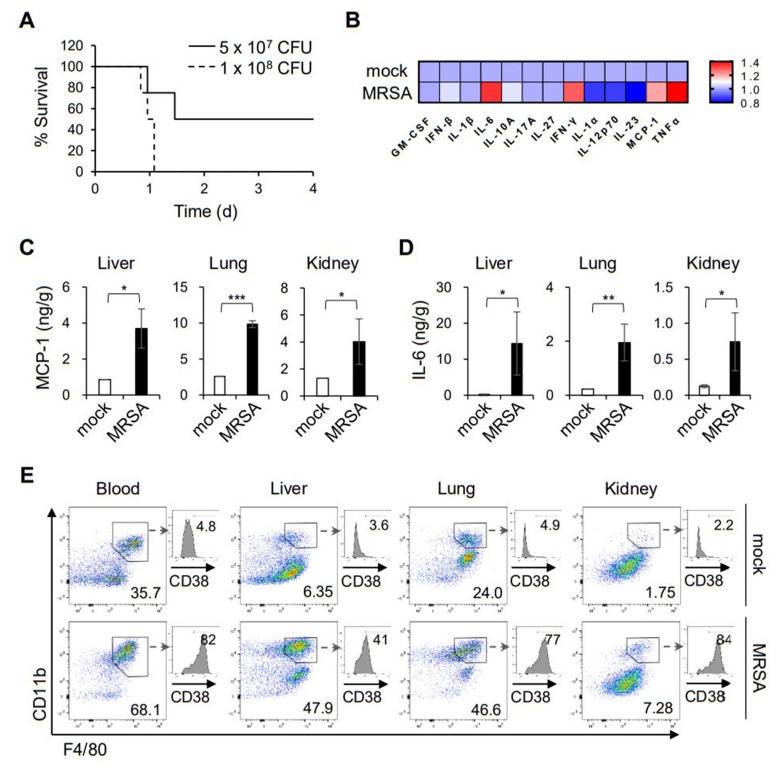
Infiltration and activation of pro-inflammatory macrophages upon MRSA infection. (**A**) Mice were infected intravenously with two different doses of MRSA, and mortality was monitored; *n* = 4. (**B**) Cytokines and chemokines in the plasma were measured by CBA 8 h after infection with 5 × 10^7^ CFU MRSA. The relative amounts of cytokines are presented as a heat map. (**C** and **D**) Tissue lysates of the liver, lung and kidney were prepared 8 h after infection, and the cytokine levels were determined by CBA. The mean (± S.D.) amounts of MCP-1 (**C**) and IL-6 (**D**) are shown; * *p* < 0.05, ** *p* < 0.01, *** *p* < 0.001. (**E**) Single cell suspension of the tissue homogenates was stained for flow cytometry, and the representative FACS profiles are shown with the frequency of the CD11b^+^ F4/80^+^ populations in the viable CD45.2^+^ gates; *n* = 3. CD11b^+^ F4/80^+^ macrophages were further analyzed for surface expression of CD38 and shown as histograms with the frequency of CD38^+^ cells.

The innate immunity against microbial infections is vital for the clearance of invading pathogens, which coincides with directing the subsequent adaptive immunity [15]. Prior to defining an immunomodulatory herbal compound, we evaluated the innate immune responses triggered by MRSA infection using an animal model. An intravenous infection of C57BL/6 mice with MRSA led to septic death, in which infection with 1 × 10^8^ CFU was sufficient to induce 100% lethality (Figure 1A). After 8 h post-infection, the production of pro-inflammatory cytokines and chemokines, including IL-6, IFN-γ, TNF-α, and MCP-1, was pronounced in the plasma (Figure 1B), which is associated with the septic symptoms. Similarly, tissue expression of MCP-1 and IL-6 was substantially induced in the liver, lung, and kidney (Figure 1C,D). Because macrophages are one of the major populations responsible for the early innate immunity against microbial infection, we examined the monocyte/macrophage populations in the tissues and peripheral blood. Flow cytometric analyses showed that CD11b^+^ F4/80^+^ cells were highly enriched in the liver, lung, kidney, and peripheral blood at 8 h post MRSA infection (Figure 1E). Moreover, those macrophages exhibited a pro-inflammatory phenotype (the so-called M1), as shown by the expression of CD38, a marker for the pro-inflammatory macrophages [16]. These results confirmed the importance of macrophages in the early immune responses against MRSA infection. 

### 2.2. DA Inhibits LPS-Induced Production of Pro-Inflammatory Cytokines in Macrophages

**Figure 2:**
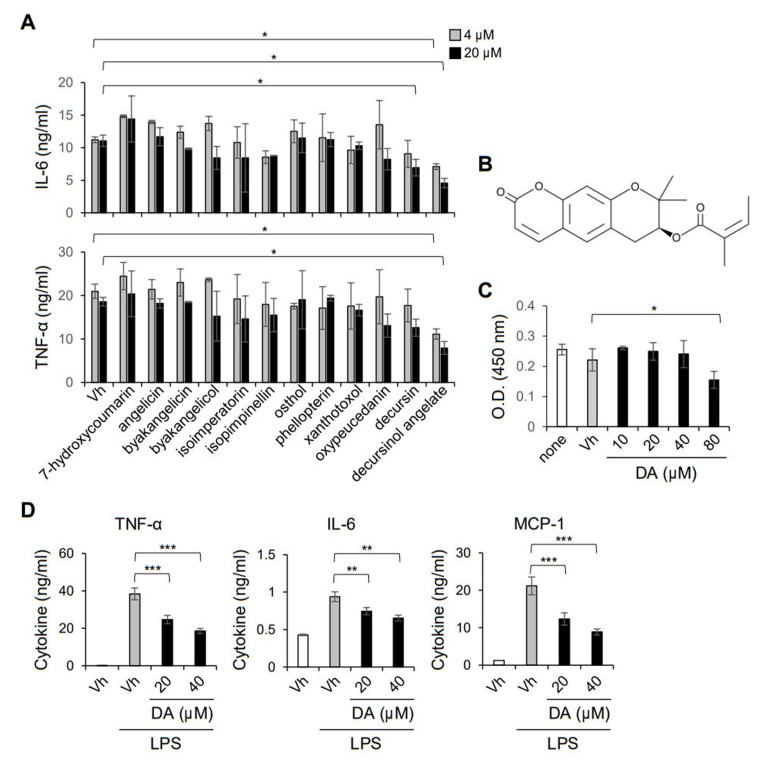
DA suppresses LPS induction of pro-inflammatory cytokines in macrophages. (**A**) Peritoneal macrophages were pretreated with the indicated compounds of *A. gigas* and activated with 200 ng/mL LPS for 8 h. Production of IL-6 and TNF-α in the culture supernatant was measured by CBA; *n* = 4. (**B**) Chemical structure of DA. (**C**) BMDMs were incubated in the presence of the indicated concentrations of DA for 24 h, and cell viability was assessed. (**D**) RAW 264.7 cells were pretreated with DA and incubated in the presence of LPS for 8 h. LPS induction of TNF-α, IL-6, and MCP-1 was determined by CBA and shown as the mean (± S.D.) concentration of the indicated cytokines; *n* = 4; * *p* < 0.05, ** *p* < 0.01, *** *p* < 0.001.

Studies have reported that the roots of *A. gigas* have potential for the treatment and prevention of inflammatory diseases, including rheumatoid arthritis and allergy [12,17]. To identify the bioactive compound of *A. gigas* that is responsible for the management of sepsis induced by pathogen infection, we first evaluated the anti-inflammatory effect of twelve major ingredients of *A. gigas* using peritoneal macrophages. Among the compounds of *A. gigas*, decursinol angelate (DA) markedly suppressed the secretion of the pro-inflammatory cytokines IL-6 and TNF-α from macrophages activated with LPS (Figure 2A,B). Although decursin was also reported to inhibit the LPS induction of inflammatory mediators, such as TNF-α in RAW264.7 and THP-1 cell lines [13], we observed a relatively modest effect of decursin on primary mouse macrophages. DA up to 40 μM did not affect the cell viability, implying that the reduced amounts of IL-6 and TNF-α were not due to the cytotoxic effect of DA (Figure 2C). The immunomodulatory effect on macrophages was further confirmed using the murine monocyte/macrophage RAW264.7 cell line. Pretreatment with DA significantly inhibited the production of TNF-α, IL-6, and MCP-1 in LPS-activated RAW264.7 cells (Figure 2D). Thus, we revealed that DA has anti-inflammatory activity to modulate the pro-inflammatory responses of macrophages in vitro.

### 2.3. DA Alleviates Septic Symptoms Induced by MRSA Infection

**Figure 3:**
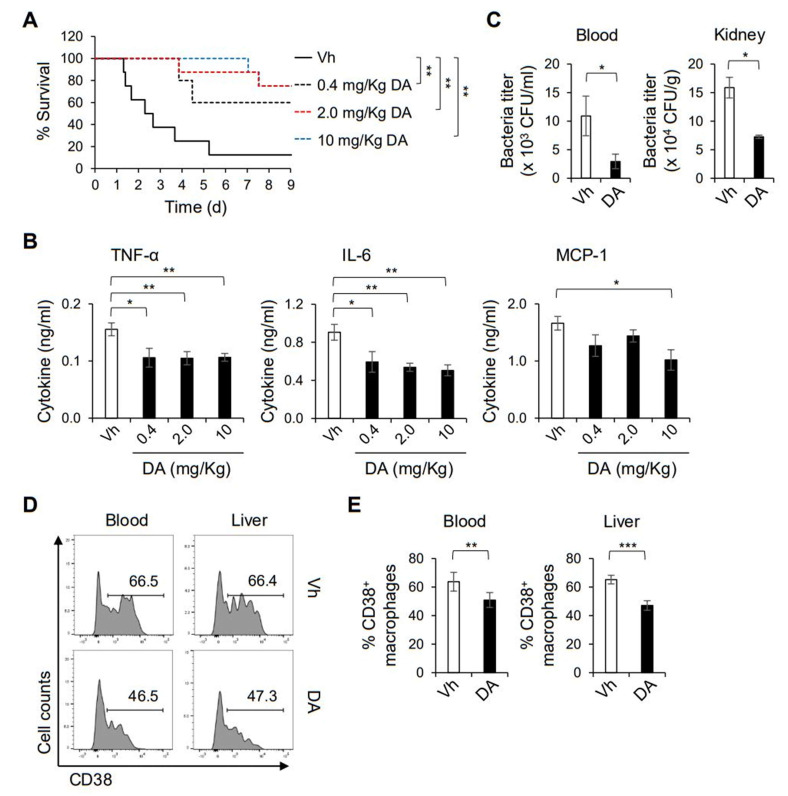
DA mitigates sepsis induced by methicillin-resistant *S. aureus* infection. (**A**) Mice were intraperitoneally pretreated with the indicated doses of DA three times and intravenously infected with 1 × 10^8^ CFU MRSA. A Kaplan–Meier plot is shown; *n* = 8. (**B**) Amounts of TNF-α, IL-6 and MCP-1 in the plasma were measured by CBA. (**C**) Bacterial titers in the blood and kidney were determined by colony-forming assay 8 h after MRSA infection; *n* = 6. (**D**,**E**) Macrophage population in the blood and liver was analyzed by flow cytometry 8 h after MRSA infection. Representative histograms with the frequencies of the CD38^+^ cells in the CD11b^+^ F4/80^+^ gates (**D**) and the mean (± S.D) frequency of CD38^+^ macrophages (**E**) are shown; *n* = 5; * *p* < 0.05, ** *p* < 0.01, *** *p* < 0.001.

To determine the anti-inflammatory potential of DA in vivo, we intraperitoneally pretreated mice with DA and infected them intravenously with a lethal dose of MRSA. As shown by the Kaplan–Meier analysis in Figure 3A, we observed a dose-dependent protective effect of DA on septic death induced by MRSA infection. The induction of proinflammatory mediators, such as TNF-α, IL-6, and MCP-1, upon MRSA infection was significantly attenuated when the mice were pretreated with DA, which could be associated with the improved septic symptoms (Figure 3B). Moreover, the bacterial titers in the kidney and peripheral blood were substantially decreased in the mice treated with DA (Figure 3C), while those in the liver and lung showed no significant difference between the mice treated with DA and the vehicle (data not shown). Intriguingly, flow cytometric analysis revealed that the cell surface induction of CD38 on CD11b^+^ F4/80^+^ cells in the liver and peripheral blood was reduced by the DA treatment, while the prevalence of the CD11b^+^ F4/80^+^ populations was similar between the mice with DA and the vehicle (Figure 3D,E). In line with the in vitro experiments (Figure 2), these results show that DA could modulate the pro-inflammatory activation of macrophages in response to pathogenic bacteria. On the other hand, we did not observe a significant difference in the proportion of CD38^+^ macrophages in the lung and kidney (data not shown). Collectively, DA mitigates septic symptoms by suppressing the production of pro-inflammation cytokines and improving bacteremia, while simultaneously altering the functional activity of macrophages. 

### 2.4. DA Enhances the Bacterial Killing Ability of Macrophages While Suppressing the Induction of Pro-Inflammatory Cytokines in Response to MRSA

**Figure 4:**
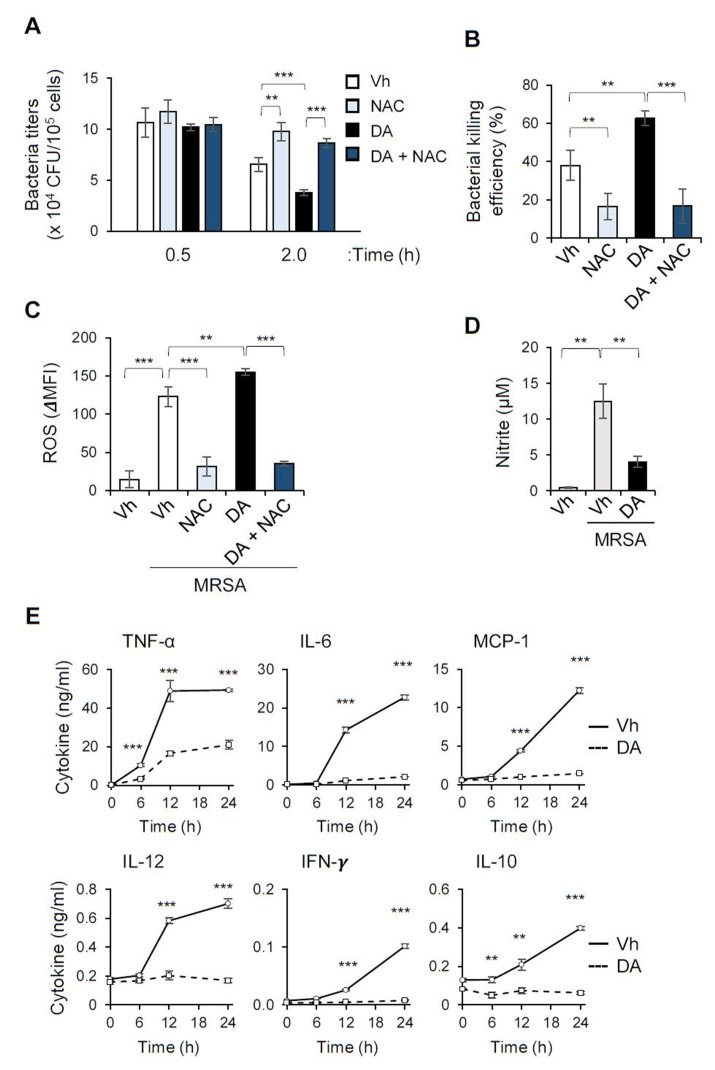
DA promotes the bacterial killing activity of macrophages while suppressing the induction of pro-inflammatory cytokines. BMDMs were pretreated with 40 µM DA and incubated with 3 × 10^6^ CFUs MRSA. Some macrophages were incubated with MRSA in the presence of 5 mM *n*-acetylcysteine (NAC). (**A**,**B**) Cells were washed to remove cell-free bacteria 0.5 h after infection with MRSA and incubated for additional 1.5 h in a new antibiotic-free medium. BMDMs were lysed, and the bacterial titer at the indicated times (0.5 h and 2.0 h) after the MRSA infection were determined as CFU; *n* = 5 (**A**). (**B**) Bacterial killing efficiency was calculated as described in the “Materials and Methods”, and the mean (± S.D.) values are shown. (**C**,**D**) BMDMs were pretreated with DA and infected with 3 × 10^6^ CFUs MRSA for 2 h in the presence or absence of NAC. ROS levels in BMDMs were measured using H_2_DCFDA and flow cytometry (**C**). Amounts of NO in the culture supernatant were determined as nitrite levels (**D**). (**E**) Cytokine production of BMDMs in response to MRSA infection was determined by CBA over a time-course; *n* = 4; * *p* < 0.05, ** *p* < 0.01, *** *p* < 0.001.

In the in vivo infection experiment, DA treatment not only attenuated the induction of pro-inflammatory cytokines upon MRSA infection, but restrained the bacterial loads in the kidney and the plasma. Macrophages are the main phagocytes of infectious agents, especially in the liver, where they are important for the dissemination of pathogens drained from the portal vein to other organs, such as the kidneys [18]. To determine the effect of DA on the phagocytic function of macrophages, we incubated bone marrow-derived macrophages (BMDMs) directly with MRSA in vitro. After a 30 min incubation with MRSA, we did not observe a significant difference in the bacterial uptake between the BMDMs pretreated with DA and the vehicle (Figure 4A). However, the live bacterial titers in the BMDMs were markedly decreased by the DA treatment, compared to the control BMDMs, when the macrophages were further incubated for 1.5 h (Figure 4A). This result suggests that DA upregulates the killing ability of macrophages, but not the earlier phagocytic uptake of invading pathogens (Figure 4B). The efficacy of the killing ability of the ingested bacteria is facilitated by phagosomal maturation and the induction of anti-microbial effectors, such as reactive oxygen species (ROS) and nitric oxide (NO) [19]. We observed robust induction of ROS and NO in BMDMs when the macrophages were incubated with MRSA (Figure 4C,D). Intriguingly, the DA treatment enhanced the MRSA-induced production of ROS, whereas it suppressed that of NO (Figure 4C,D). These results imply a distinct impact on the production of antimicrobial effector molecules such as ROS and NO. To further validate the contribution of ROS to the increased bactericidal activity, we incubated macrophages with N-acetylcysteine (NAC), a ROS scavenger [20], and observed substantially reduced ROS production induced by MRSA (Figure 4C). NAC abrogated macrophage’s ability to kill the ingested bacteria, and DA treatment did not enhance bactericidal activity in the presence of NAC (Figure 4A,B). This result confirmed that ROS is one of the major effectors to kill bacteria uptaken by macrophages, and suggested that the enhanced killing ability by DA would be due to increased ROS production.

Infection of the BMDMs with MRSA robustly upregulated the secretion of an array of inflammatory mediators that included TNF-α, IL-12, IFN-γ, and IL-10 (Figure 4D). Even in this robust activating condition, DA potently inhibited the production of the inflammatory cytokines in the macrophages (Figure 4D). These data indicate that DA can modulate the functional activity of macrophages against MRSA, which might contribute to the improved bacteremia and cytokine storm in mice infected with MRSA.

### 2.5. DA Modulates the Functional Activation of Macrophages 

**Figure 5:**
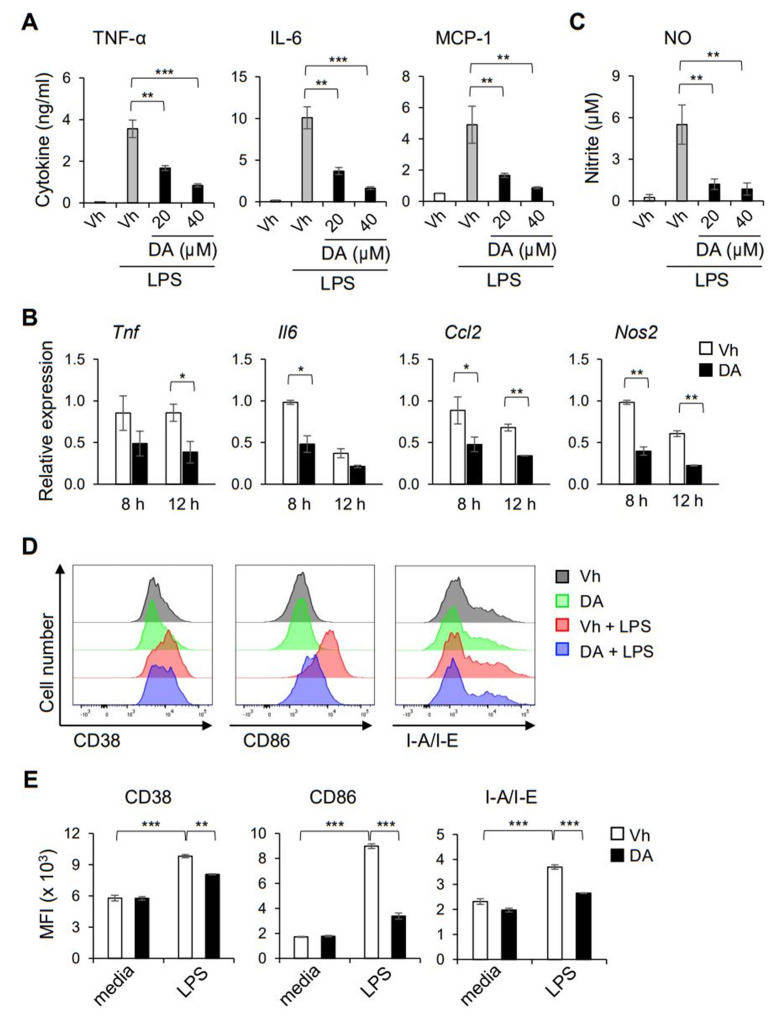
DA inhibited the LPS-induced functional activation of pro-inflammatory macrophages. (**A**) BMDMs were pretreated with the indicated concentrations of DA and activated with 200 ng/mL LPS for 8 h. Production of TNF-α, IL-6 and MCP-1 in the culture supernatant was determined by CBA; *n* = 4. (**B**) Amounts of nitrite in the culture supernatant were measured. (**C**) BMDMs were pre-treated with 20 µM of DA followed by stimulation with LPS for the indicated time. LPS-induced expression of mRNA encoding the indicated genes was quantitated by real-time PCR; *n* = 3. (**D**,**E**) BMDMs were pretreated with 20 µM DA and activated with LPS for 24 h. Cell surface expression of CD38, CD86 and I-A/I-E was analyzed by flow cytometry. Representative histograms (**D**) and the mean (± S.D.) MFI of the indicated surface markers (**E**) are shown; *n* = 3; * *p* < 0.05, ** *p* < 0.01, *** *p* < 0.001.

To further define the immunomodulatory effect on the functional activation of macrophages, we determined whether DA could regulate the pro-inflammatory (so-called ‘M1′) activation of macrophages triggered by LPS. Consistent with its effect on the peritoneal macrophages shown in Figure 2A, pretreatment with DA suppressed the LPS-induced production of TNF-α, IL-6, and MCP-1 in the BMDMs (Figure 5A). Quantification of mRNA showed that the expression of genes encoding those pro-inflammatory mediators was decreased by the DA treatment (Figure 5B), indicating regulation at the transcriptional level. DA also inhibited the production of NO in BMDMs activated with LPS (Figure 5C), which was associated with decreased expression of the gene encoding iNOS (Figure 5B). Activation of macrophages with LPS stimulation upregulates cell surface markers that are associated with pro-inflammatory activity [21]. Flow cytometry analysis revealed that DA attenuated the LPS-induced surface expression of a costimulatory ligand CD86, class II MHC (I-A/I-E), and CD38 on the BMDMs (Figure 5D,E). Nonetheless, the DA treatment did not upregulate the surface induction of markers associated with the alternatively activated macrophages (the so-called ‘M2′), including CD206 and CD273 (data not shown). Consistently, the production of the anti-inflammatory cytokine IL-10 was not upregulated, but decreased by DA, as shown in Figure 4D. Overall, these results show that DA has an immunomodulatory potential to suppress the functional polarization of pro-inflammatory macrophages, but not to promote that of immunoregulatory macrophages.

### 2.6. DA Suppressed the NF-κB Pathway Independently of Akt Inhibition

**Figure 6:**
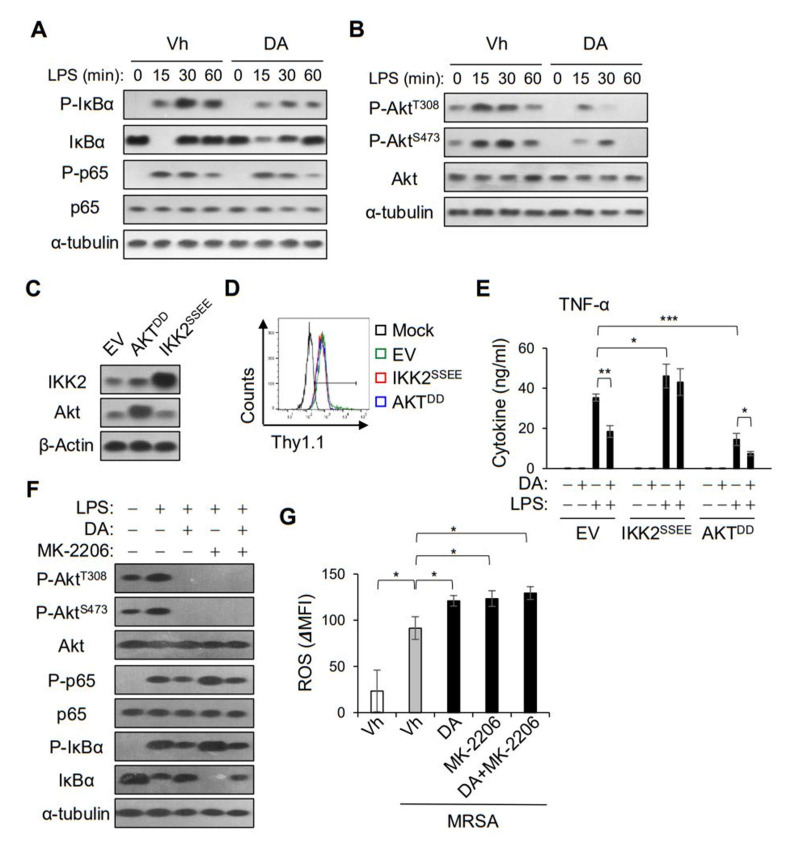
DA modulates NF-κB and Akt signaling pathways. (**A**,**B**) BMDMs were pretreated with 20 µM DA followed by LPS stimulation over a time-course and analyzed by Western blotting with the indicated antibodies. Representative immunoblots from three independent experiments are shown. Immunoblotting for α-tubulin and unphosphorylated proteins was used as a loading control. (**C**–**E**) RAW 264.7 cells were transfected with the indicated plasmids, and ectopic expression of IKK2^SSEE^ and Akt^DD^ was confirmed by Western blotting (**C**) and flow cytometry (**D**). Cells were pretreated with DA, and secretion of TNF-α was determined 8 h after LPS activation (**E**); *n* = 3. (**F**,**G**) BMDMs were pretreated with 20 µM DA, 1 µM MK-2206, or both. One hour after LPS stimulation, the cells were analyzed by Western blotting with the indicated antibodies, and ROS production was determined by flow cytometry; * *p* < 0.05, ** *p* < 0.01, *** *p* < 0.001.

To investigate the molecular mechanism by which DA regulates the inflammatory responses of macrophages, we performed a biochemical analysis of the cellular signaling involved in the expression of pro-inflammatory mediators. The MAPK and NF-κB signaling pathways promote the expression of pro-inflammatory cytokines, such as TNF-α, via the transcriptional activation of the AP-1 and NF-κB dimers, respectively [22]. While the MAPK pathway was marginally affected, as shown by the phosphorylation of Erk1/2 and p38 (Supplemental Figure S1), the LPS-induced phosphorylation of IκBα and NF-κB p65 was apparently attenuated by the DA treatment in the BMDMs (Figure 6A). Intriguingly, DA also inhibited the phosphorylation of Akt induced by LPS stimulation (Figure 6B). To evaluate the contributions of the NF-κB and Akt signaling pathways with the DA treatment, we transfected RAW264.7 cells with constitutively active forms of IKK2 and Akt, and measured the secretion of TNF-α (Figure 6C,D). The RAW264.7 cells expressing an active mutant of IKK (IKK2^SSEE^) produced more TNF-α than the cells transfected with the control vector (EV), confirming the importance of the NF-κB pathway in the expression of TNF-α (Figure 6E). In contrast to the results of the control cells, DA could not inhibit the LPS induction of TNF-α in cells transfected with IKK2^SSEE^ (Figure 6E). Thus, we proposed that the attenuation in the NF-κB signaling pathway in the production of proinflammatory cytokines could be perturbed by DA. On the other hand, the LPS induction of TNF-α was less efficient in cells expressing a constitutively active mutant of Akt (Akt^DD^) than in cells transfected with EV, and DA was still able to attenuate the LPS induction of TNF-α (Figure 6E). To further delineate the impact of DA on Akt signaling, we pretreated the BMDMs with MK-2206, an Akt inhibitor. In the presence of MK-2206, the phosphorylation of Akt was completely abolished, whereas the LPS-induced phosphorylation of IκBα and NF-κB p65 was slightly upregulated (Figure 6F). However, DA could inhibit the LPS-induced phosphorylation of IκBα and NF-κB p65 in cells pretreated with MK-2206 (Figure 6F). While MK-2206 treatment did not affect the production of TNF-α (data not shown), it enhanced the LPS-induced ROS production in macrophages, similarly to the DA treatment, although there was no additive effect with DA (Figure 6G). Taken together, the action of DA on the NF-κB and Akt signaling pathways might differentially modulate the functional activities of macrophages. 

## 3. Discussion

The medicinal herbal plant *A. gigas* has been widely used in East Asian traditional medicine, due to its beneficial effects on gynecological health. Studies have reported its pharmacological potential in many chronic diseases that include several types of cancer, metabolic diseases, such as diabetes, and neurological disorders, such as Alzheimer’s disease [23]. In vitro studies showed that ethanol extracts of *A. gigas* roots suppressed the production of pro-inflammatory mediators in monocytes and macrophages [13]. In addition, *A. gigas* extracts were reported to improve autoimmune diseases in rheumatoid arthritis and allergy models [12,17]. Given the anti-inflammatory potential of *A. gigas*, we identified DA as the bioactive chemical ingredient of *A. gigas* that targets the inflammatory activities of macrophages. Using bone marrow-derived and peritoneal macrophages, we observed that DA suppressed the induction of pro-inflammatory cytokines, such as TNF-α and IL-6, and surface markers associated with the functional activation of pro-inflammatory macrophages, including CD38, CD86, and class II MHC. Furthermore, DA exhibited therapeutic potential to improve septic symptoms, including mortality, and the cytokine storm in the MRSA infection model. These results suggest a modulatory effect of DA on the functional responses of macrophages with pro-inflammatory activities. 

Decursin and DA are the major bioactive components of the ethanol extracts of *A. gigas* roots, and their biological activities have been reported in several in vitro studies [12]; for example, decursin inhibited the production of NO in BV-2 microglial cells and the expression of MMP-9 in RAW264.7 [13]. DA was also found to suppress the induction of IL-1β, IL-6, and iNOS in RAW264.7 and HL-60 cells activated with LPS and phorbol-12-myristate-13-acetate, respectively [14]. In addition, we previously reported the anti-inflammatory effect of DA on T cells, in which DA suppressed type 17 helper T-cell responses and ameliorated colitis in a mouse model [8]. However, the biological impact of decursin and DA was unclear on the functional activities of primary macrophages and/or macrophage-associated inflammatory diseases. In this study, we showed the bioactive potential of DA on mouse peritoneal and bone marrow-derived macrophages, and on tissue macrophages, in a septic animal model. In accordance with the previous studies, DA potently inhibited the production of proinflammatory cytokines, such as IL-6 and TNF-α, and the induction of NO in BMDMs stimulated with LPS or MRSA bacteria. Intriguingly, DA promoted the bacterial killing ability of macrophages, while it did not affect the phagocytic function (i.e., bacterial uptake). This explains the in vivo data, in which the bacterial titers in the plasma and liver of mice infected with MRSA were reduced by the DA treatment. 

Upon pathogen infection, macrophages engulf the invading bacteria by phagocytosis, and the phagosomes later fuse with lysosomes, eventually killing the bacteria [19]. During this process, the macrophages undergo a respiratory burst to produce ROS and NO that eliminate the bacteria. Indeed, DA treatment increased the ROS production in macrophages infected with MRSA, although it suppressed NO synthesis. The respiratory burst is upregulated in activated macrophages with the pro-inflammatory phenotype, and has a major role in eliminating the phagocytosed bacteria [19]. Similarly, Canton et al. showed increased phagolysosome formation in macrophages with immunomodulatory activities, and decreased phagolysosomes in pro-inflammatory macrophages [24]. We also observed impaired bactericidal activity when treated with ROS scavenger NAC (Figure 4), confirming that ROS is one of the major effectors to kill pathogenic microbes uptaken by macrophages. Intriguingly, the inhibition of ROS abrogated the enhanced killing ability by DA. Thus, we speculated that the enhanced bactericidal activity by DA treatment might be due to the increased production of ROS, although we could not exclude the possibility that DA regulates phagosome maturation and lysosomal fusion.

Biochemical analysis showed that DA attenuated the LPS-induced phosphorylation of IκBα and NF-κB p65, which regulate the transcription of many inflammatory genes, including *Il1b*, *IL6*, and *TNF*. On the other hand, we observed a relatively modest effect on the LPS-induced phosphorylation of ERK-1/2 and p38, although DA was reported to modulate MAPK signaling in studies using cell lines or cancer cells [14,25]. Of note, the enforced expression of a constitutively active form of IKK2 enhanced the expression of TNF-α and abrogated the inhibitory effect of DA, suggesting that the anti-inflammatory action of DA might be largely due to its modulation of the transcriptional activity of NF-κB signaling. DA also inhibited the phosphorylation of Akt, triggered by the LPS treatment and MRSA infection. PI3K-Akt signaling is essential for the survival and growth of macrophages, and is reported to be involved in activated macrophages with a regulatory phenotype [26]. We observed that the expression of the constitutively active form of Akt was not able to enhance the LPS induction of TNF-α, but negatively regulated it. Rather, the inhibition of Akt by MK-2206 increased the ROS production in BMDMs infected with MRSA. These results imply that the action of DA on Akt signaling could be associated with the bactericidal activity of macrophages.

To define the anti-inflammatory potential of DA on a disease condition primarily associated with macrophages, we used the acute sepsis model with MRSA, a major serious threat, especially in hospitals [6]. The deregulated activation of macrophages with pro-inflammatory phenotypes upon MRSA infection is responsible for the cytokine storm and tissue damage, leading to fatal septic shock [27,28]. Indeed, lethal infection with MRSA upregulated the production of IL-6, TNF-α, and MCP-1 in the circulation and organs such as the liver, lungs, and kidneys, which was correlated with the induction of CD38 associated with pro-inflammatory macrophages (Figure 1). In agreement with the in vitro results, DA treatment attenuated the production of TNF-α and IL-6 in the plasma, and improved mortality in a dose-dependent manner. As far as we know, no toxic symptoms have been reported for decursin or DA up to dose of 200 mg/Kg in rats following oral administration, and at a dose range from 10 to 100 mg/Kg in mice after intraperitoneal administration multiple times [29,30,31]. The pharmacokinetic study also showed that DA was rapidly absorbed from the gastrointestinal tract after oral administration, and its metabolites are primarily excreted into feces, with no notable accumulation in the tissues and whole blood [29]. Thus, we speculate that DA might have a good balance between efficacy and safety as a natural anti-inflammatory compound, at least in considering the dose range (from 0.4 to 10 mg/Kg) used in our infection experiment.

Macrophages with the pro-inflammatory phenotype were also decreased by DA, as shown by the downregulation of CD38^+^ populations in the plasma and liver, by which MRSA is primarily sequestered from the portal vein, and then disseminated to other organs, such as the kidneys [18]. Vancomycin and daptomycin are currently used for the management of patients with MRSA infection, and combinations with alternative antibiotics have been tested [32]. Although antibiotics are the primary option for the treatment of sepsis, their excessive use has resulted in the increasing emergence of antibiotic-resistant strains worldwide [33]. Because there is an urgent need for an alternative management of sepsis triggered by antibiotic-resistant pathogenic strains, our study highlights a natural compound that can suppress the cytokine storm and sepsis symptoms by modulating the pro-inflammatory activation of macrophages. Moreover, it would be challenging to develop an alternative approach to treat sepsis in a combination of targeting the phagocytic activity of macrophages with low doses of antibiotics.

## 4. Materials and Methods

### 4.1. Reagents

Twelve compounds of *A. gigas* including decursinol angelate were provided by Yoongho Lim (Konkuk University, Seoul, Korea); the purity of each compound was at least 98% which was determined by a HPLC system fitted with an RP-C18 column (Phenomenex, Torrance, CA, USA) [34]. Recombinant murine macrophage colony-stimulating factor (M-CSF) was purchased from PeproTech (Cranbury, NJ, USA) and recombinant murine IL-4 from R&D systems (Minneapolis, MN, USA). Lipopolysaccharide (LPS) and N-acetylcysteine (NAC) were obtained from Sigma Aldrich (St. Louis, MO, USA). MK-2206 was purchased from Selleck (Houston, TX, USA). Primary antibodies against α-tubulin, phosphor-p65, p65, phospho-IκBα, IκBα, phosphor-Akt^S437^, phospho-AktT^308^, Akt, phospho-Erk1/2, Erk1/2, phospho-p38 and p38 were obtained from Cell Signaling Technologies (Danvers, MA, USA). Fluorophore-conjugated antibodies against CD11b (clone: M1/70), F4/80-like receptor (clone: 6F12), CD274 (clone: MIH5), I-A/I-E (clone: M5/114.15.2) and CD86 (clone: Gl-7) were purchased from BD Biosciences (San Jose, CA, USA), except the PE-conjugated CD38 (clone: 90) antibody (eBioscience, San Diego, CA, USA).

### 4.2. Bacteria Culture and Colony Formation Assay

MRSA (MW2 strain) was cultured overnight in 3.5% Columbia broth (BD Difco, Franklin Lakes, NJ, USA) supplemented with 2% NaCl in a 37 °C shaking incubator [35]. Then, the bacterial culture was diluted in fresh media at 1:50 and grown to an OD_600_ of 0.5 which is equivalent to 3 × 10^8^ CFUs/mL. The bacterial culture was washed twice and resuspended in PBS prior to infection. To measure the colony formation units, tissue homogenates, blood collected from the tail vein, or macrophage lysates were diluted serially in PBS and spread onto Columbia broth plates containing 1.5% agar with the help of plastic beads. Colonies grown on the plates were counted 12 h after incubation at 37 °C, and the CFUs per gram of tissue or per milliliter of blood were determined.

### 4.3. Mice

C57BL/6J mice were purchased from DBL (Seoul, Korea). All animal experiments were conducted in accordance with the guidelines and experimental protocols approved by the Institutional Animal Care and Use Committee of Hallym University (Hallym 2018-57). After 72 h of acclimation in an animal biosafety level 2 facility, seven- to eight-week-old mice were intraperitoneally administered the indicated doses of DA 3 times every alternate day and then intravenously infected with 5 × 10^7^ or 1 × 10^8^ CFUs of MRSA. At 8 h post-infection, the liver, lung, and kidney were isolated after cardiac perfusion and weighed followed by mechanical homogenization [36]. Briefly, the organs were immersed in 1 mL PBS and homogenized by steel beads using TissueLyser II (QIAGEN, Hilden, Germany). For cytokine measurements, the homogenates were centrifuged at 13,000 rpm for 10 min at 4 °C, and the supernatants were analyzed by cytometric bead array (CBA) (BD Biosciences, San Jose, CA, USA). 

### 4.4. Macrophages

Mice were intraperitoneally treated with 3% thioglycolate for 4 days, and peritoneal lavage was collected by injecting ice-cold PBS into the peritoneum. Peritoneal cells were washed, counted and resuspended in IMDM media containing 10% FBS, 100 units/mL penicillin, 100 µg/mL streptomycin and 0.1 mM β-mercaptoethanol. After waiting for at least 2 h, enabling adherence to a well plate, non-adherent cells were removed by changing the medium. The adherent peritoneal macrophages were pretreated with the indicated herbal compounds for 1 h and activated with 200 ng/mL LPS cells for 8 h. Bone marrow-derived macrophages (BMDMs) were prepared as described previously [37]. Briefly, bone marrow cells were isolated from femurs and tibias and cultured for 7 days in a Petri dish in the presence of 20 ng/mL M-CSF. BMDMs were harvested by adding non-enzymatic cell dissociation solution (Corning, Tewksbury, MA, USA) to the Petri dish and seeding them into a well-plate. RAW264.7 cells were provided by Hyung-Joo Kwon (Hallym University, Chuncheon, Korea) and cultured in DMEM supplemented with 10% FBS. RAW264.7 cells were transfected with MSCV-Akt(DD)-IRES-Thy1.1 [AKT^DD^], MSCV-IKK2(SSEE)-IRES-Thy1.1 [IKK2^SSEE^], or control vector [EV] using the FuGene^®^ HD transfection reagent (Promega, Madison, WI, USA) following the manufacturer’s instructions. 

### 4.5. Cell Viability Assay

BMDMs were cultured in the presence of DA for 24 h, and cell viability was assessed using the EZ-Cytox enhanced cell viability assay kit (DoGenBio, Seoul, Korea) following the manufacturer’s instruction.

### 4.6. Flow Cytometry and Cytokine Measurement

A single cell suspension was prepared as described elsewhere [38]. The liver, lung and kidneys were harvested after cardiac perfusion and cut into pieces followed by incubation at 37 °C for 30 min with the following digestion buffers: 25 μg/mL of Liberase DL, 25 μg/mL of Liberase TL (Roche, Basel, Switzerland) and 125 μg/mL of DNase I (Sigma, St. Louis, MO, USA) in serum-free RPMI media. Then, EDTA was added to a final concentration of 1 mM and incubated at 4 °C for 10 min followed by passing through a 70 μm cell strainer. After the cells were incubated with RBC lysis buffer, the viable cells were counted and resuspended in FACS wash buffer (1% FBS in PBS). Single cell suspensions isolated from the liver, lung and kidney, and in vitro cultures, were stained with fluorescently labelled antibodies against surface markers for 30 min on ice. After washing and resuspending in FACS buffer (1% FBS in PBS), the cells were analyzed by the FACS Canto II instrument (BD Biosciences) with the FlowJo V10 software. For intracellular staining of CD206, BMDMs were fixed with 4% paraformaldehyde, permeabilized with 1% saponin in FACS buffer, and stained with APC-conjugated anti-CD206 antibodies. Macrophages were presented as CD11b^+^ F4/80^+^ cells in a viable FSC and SSC gate. Amounts of cytokine levels in the tissue homogenates, plasma and culture supernatants were measured using the BD CBA mouse inflammatory cytokine kit (BD Biosciences) and LEGENDplex^TM^ mouse inflammation panel (BioLegend, San Diego, CA, USA) in accordance with the manufacturer’s instructions.

### 4.7. Phagocytosis and Bacteria-Killing Assay

BMDMs were pretreated with 40 μM DA for 1 h and incubated with 3 × 10^6^ CFUs of MRSA in antibiotic-free IMDM. Some cells were incubated with MRSA in the presence of 5 mM N-acetylcysteine (NAC). Then, 30 min after the incubation, the remaining cell-free bacteria were washed extensively with PBS, and the BMDMs were lysed immediately (phagocytosis assay) or after 90 min of further incubation in fresh media (bacteria-killing assay) using 0.2% Triton X-100 [39]. The amounts of live bacteria were determined by the colony formation assay on Columbia broth agar plates. The killing efficiency was calculated as $\frac{A-B}{A}\times 100\;\%\;$(where A = bacteria titer in the phagocytosis assay and B = bacteria titer in the killing assay). 

### 4.8. Measurement of Reactive Oxygen Species and Nitric Oxide

BMDMs were pre-treated with DA for 1 h and activated with 200 ng/mL LPS or 3 × 10^6^ CFUs of MRSA for 2 h. Production of reactive oxygen species was measured by detecting the oxidation of 2′,7′-dichlorodihydrofluorescein diacetate (H_2_DCFDA) [40]. Briefly, BMDMs were incubated at 37 °C with 5 μM H_2_DCFDA (Thermo Fisher Scientific) for 20 min and analyzed by flow cytometry. The nitrite content in the culture supernatant was measured by the Griess reagent system (Promega, Madison, WI, USA) following the manufacturer’s instructions.

### 4.9. Quantitative Real-Time PCR

Total RNA was extracted using the TRIZOL reagent (Thermo Fisher Scientific, Waltham, MA, USA) and reverse transcribed into cDNA by the ImProm-II reverse transcription system kit (Promega, Madison, QI, USA). Using the primer pairs listed in Supplemental Table S1, quantitative real-time PCR was performed with the SYBR qPCR mix (Toyobo, Osaka Japan) and the CFX96 real-time PCR detection system (Bio-Rad, Hercules, CA, USA). Data were normalized to the levels of *Actb* in each sample and quantitated by the comparative 2^ΔΔCt^ method.

### 4.10. Western Blotting

Cells were lysed in lysis buffer containing 20 mM HEPES (pH 7.4), 150 mM NaCl and 1% TritonX-100 supplemented with a protease inhibitor cocktail and phosphatase inhibitor cocktail (GenDEPOT, Baker, TX, USA). Equal amounts of lysates were resolved in SDS-PAGE and transferred onto the PVDF membrane. The membrane was incubated with the indicated primary antibody in 5% skim milk for 2 h followed by incubation with the HRP-conjugated secondary antibody for 1 h. Protein bands were detected on X-ray film using an enhanced chemiluminescence kit (Merck Millipore, Darmstadt, Germany).

### 4.11. Statistical Analysis

Experiments were performed in triplicate, and the results of at least three independent experiments are represented as the mean ± S.D. Statistical analysis was conducted using unpaired Student’s *t*-test (Instat; GraphPad Inc., San Diego, CA, USA). The Kaplan–Meier log-rank test was performed using R (The R Foundation for Statistical Computing, Vienna, Austria). A *p*-value less than 0.05 was considered to be statistically significant.

## Supplementary Materials

The following are available online at https://www.mdpi.com/article/10.3390/ijms222010950/s1: Figure S1: effect of DA on the MAPK signaling pathway; Table S1: primer sequences used in the quantitative real-time PCR analysis.
