# Supplementary material for: Decursinol Angelate Mitigates Sepsis Induced by Methicillin-Resistant Staphylococcus aureus Infection by Modulating the Inflammatory Responses of Macrophages
Source: Int J Mol Sci. 2021 Oct 11;22(20):10950. doi: 10.3390/ijms222010950 (PMC8539957; doi:10.3390/ijms222010950)

SUPPLEMENTAL INFORMATION

Decursinol angelate mitigates sepsis induced by methicillin-resistant *Staphylococcus aureus* infection by modulating the inflammatory responses of macrophages

Seongwon Pak[1, 3], Bikash Thapa[2, 3] and Keunwook Lee[1, 2, *]

[1]Department of Biomedical Science and [2]Institute of Bioscience and Biotechnology, Hallym University, Chuncheon, 24252, Korea; [3] These authors contributed equally to this work

* Correspondence: K.L.; Email. keunwook@hallym.ac.kr; Phone. +82-33-248-2113

Supplemental Table S1. Primer sequences used in the quantitative real-time PCR analysis

Gene name	Sequence (5' to 3')
Tnf	GATCGGTCCCCAAAGGGATG<br>CACTTGGTGGTTTGCTACGAC
Il6	GTTCTCTGGGAAATCGTGGA<br>TGTACTCCAGGTAGCTATGG
Ccl2	AACTGCATCTGCCCTAAGGT<br>AGGCATCACAGTCCGAGTCA
Nos2	TCCTGGACATTACGACCCCT<br>CTCTGAGGGCTGACACAAGG
Actb	GGCACCACACCTTCTACAATG<br>GGGGTGTTGAAGGTCTCAAAC

Supplemental Figure Legend

Supplemental Figure S1. Effect of DA on the MAPK signaling pathway. BMDMs were pretreated with 20 μM DA followed by LPS stimulation over a time-course and analyzed by Western blotting with the indicated antibodies. Representative immunoblots from 2 independent experiments are shown. Immunoblotting for α-tubulin and unphosphorylated proteins was used as a loading control.

Pak *et al*. Supplemental Figure S1

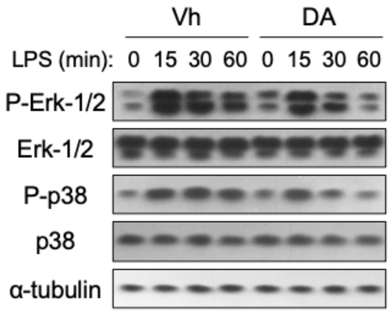

Supplement: Supplementary file 1 [file ijms-22-10950-s001.zip › ijms-1395871-SI.pdf]
